# Liver and Steroid Hormones—Can a Touch of p53 Make a Difference?

**DOI:** 10.3389/fendo.2019.00374

**Published:** 2019-06-12

**Authors:** Meital Charni-Natan, Ronit Aloni-Grinstein, Etty Osher, Varda Rotter

**Affiliations:** ^1^Department of Molecular Cell Biology, Weizmann Institute of Science, Rehovot, Israel; ^2^Department of Biochemistry and Molecular Genetics, Israel Institute for Biological Research, Ness-Ziona, Israel; ^3^Sackler Faculty of Medicine, Tel Aviv-Sourasky Medical Center, Institute of Endocrinology Metabolism and Hypertension, Tel Aviv University, Tel Aviv, Israel

**Keywords:** p53, steroid hormones, liver, NAFLD, cirrhosis, estrogen, testosterone

## Abstract

The liver is the main metabolic organ in the body, serving as a significant hormonal secretory gland and functioning to maintain hormone balance and homeostasis. Steroid hormones regulate various biological pathways, mainly in the reproductive system and in many metabolic processes. The liver, as well as steroid hormones, contribute significantly, through functional intertwine, to homeostasis maintenance, and proper responses during stress. Malfunction of either has a significant impact on the other and may lead to severe liver diseases as well as to several endocrine syndromes. Thus, the regulation on liver functions as on steroid hormones levels and activities is well-controlled. p53, the well-known tumor suppressor gene, was recently found to regulate metabolism and general homeostasis processes, particularly within the liver. Moreover, p53 was shown to be involved in steroid hormones regulation. In this review, we discuss the bi-directional regulation of the liver and the steroid hormones pointing to p53 as a novel regulator in this axis. A comprehensive understanding of the molecular mechanisms of this axis may help to prevent and treat related disease, especially with the increasing exposure of the population to environmental steroid hormones and steroid hormone-based medication.

## Introduction

Steroid hormones are involved in regulation of different biological pathways mainly in the reproductive system and in metabolic homeostasis maintenance. Steroid hormones are derived from cholesterol and are synthesized primarily by endocrine glands such as the adrenal cortex, gonads (testes and ovaries), and placenta. They are classified to five main groups, based on their corresponding nuclear receptor: mineralocorticoids, glucocorticoids, androgens, estrogens, and progestogens (1–5). The nuclear steroid receptors act as ligand-activated transcriptional activators, which may regulate different genes expression (6). Steroid hormones affect the entire body homeostasis. Thus, it is not surprising that their regulation is complex and based on several parameters including production and degradation, activation vs. inactivation and the proportion between free and bound circulating steroid compounds (7, 8).

Furthermore, this multi-level regulation takes place not only in steroidogenic tissues but also in different peripheral organs, mainly in the liver. The liver, as a central metabolic organ, plays a crucial role in steroid hormones homeostasis (9, 10) and in the elimination of toxic metabolites, which may be destructive to the tissue and in the end to the whole body, leading to ongoing stress and liver diseases. The fact that there is a tight relationship between liver pathologies and steroids hormones (Table 1), together with the enormous potential held in the use of hormone therapy for different diseases, raises the necessity of a better understanding the mechanisms underlying the liver regulation of steroid hormone balance and function. Interestingly the transcription factor p53, the well-known guardian of the genome, which reacts upon stress signals to maintain genome fidelity, was also suggested to control the entire organism homeostasis (11, 12), and to regulate many processes in the liver including steroid hormone regulation (13, 14).

**Table 1:** Steroid hormone deregulation and liver pathologies.

**Steroid status**	**Clinical condition**	**Liver disease**
Hypercortisolism	Cushing's syndrome/disease	NAFLD
Hyperaldosteronism	Primary/secondary hyperaldosteronism	NAFLD
RAAS activation	–	Liver inflammation and fibrosis
Hyperandrogenism female	Polycystic ovary syndrome	Alteration in liver metabolic homeostasis
Hyporandrogenism male (reduced testosterone)	Hypogonadism	Alteration liver metabolic homeostasisIncreased risk HCC
Hypoestrogenism	Menopause	NAFLD

*Deregulation of steroid hormone levels or bioactivity leads to various liver pathologies/diseases*.

In this review, we discuss the interesting bidirectional relationship between the different steroid hormones and the liver, independently and dependently on p53, in the context of health and liver diseases.

### Steroid Hormones Homeostasis Regulation by the Liver

The liver participates in most steps of steroid hormone regulation starting from biosynthesis of cholesterol, which is the main source for steroids biosynthesis. Cholesterol is obtained either by *de-novo* production, hydrolysis of stored cholesterol, interiorization of plasma membrane cholesterol, or from LDL and HDL, which are secreted from the liver to the plasma (15). Subsequently, the cholesterol is processed to steroids by several enzymatic steps, which occur mainly in steroidogenic organs. Further, metabolism of these steroids also occurs in the liver. For example, the liver enzyme 5α-reductase 1 (5αR1) regulates metabolic processes of both androgens and glucocorticoids (GC) (16, 17). Studies have shown that 5αR1-KO mice exhibited augmented mRNA levels of various hepatic metabolic regulators genes (e.g., *Acc1, Agpat2, Cpt2*, and *Dgat2)* in comparison to WT mice, suggesting a crucial role of the liver enzyme, 5αR1, in the regulation of GC and androgens accumulation and effects (18).

Another example is the cytochromes P450 enzymes (CYP), which are responsible for the metabolism of many drugs and lipophilic compounds (19). CYP3A4, CYP19, CYP2C2B1, and CYP2C11 are the liver CYPs that take part in steroid hormones hydroxylation and processing. CYP3A4 hydroxylases several steroids such as cortisol, androstenedione, testosterone, and progesterone (20, 21), CYP19 (Aromatase) transforms androgens to estrogens by the removal of C19 carbon and the aromatization of the steroid A ring, while CYP2C11 and CYP2B1 regulate hydroxylation of testosterone (10, 22). Accumulating data suggest that different mechanisms underlay liver regulation on CYP expression levels. Several nuclear receptors complexes such as PXR, VDR, RXR were also found to bind CYP3A4 chromatin and affect its expression (23, 24).

Additional processes such as steroids conjugation, are exerted by specific enzymes such as sulfotransferases and the uridine diphosphate-glucuronosyltransferases (UGT) that transfer the steroid hormones into higher polarity metabolites that are better suited to be excreted from the body (10). The sulfotransferases that are expressed by hepatic cells and are related to steroids conjugations are HSST, EST, SULT 2A1, and SULT 1E1 (25). Studies have shown that sulfotransferase inhibition, as well as EST KO, led to the acceleration of free steroids and thus to sexual abnormalities (10, 26). Interestingly, the sulfotransferase expression was found to be regulated by androgens, GCs, and nuclear receptors such as PXR (27), suggesting a possible regulatory feedback between the hepatic enzymes that are part of the steroid hormones processing and the steroid hormones themselves.

UGT enzymes consist of two subfamilies; the UGT2B subfamily is mainly expressed in the liver and is related to the processing of steroid hormones (28). UGT enzymes induce glucuronidation of steroids, a process that interrupts steroids activity, and enables their elimination. UGTs enzymes are regulated by several xenobiotics compounds (e.g., PCN, PB), which were reported to increase their mRNA expression levels in rats' livers (29).

As steroids, these hormones are lipophilic thus, when secreted into the blood stream they need to be bound to carrier proteins such as Sex hormone binding globulin (SHBG), Corticosteroid binding globulin (CBG) and to lesser extent albumin, which facilitate their transportation to their target organs. These carriers are glycoproteins which are secreted mainly by the liver and bind with high affinity cortisol, testosterone, and estradiol (30, 31). In addition to their role as carrier proteins, they serve as a buffer for steroid hormones homeostasis and balance. The steroid hormones binding proteins have a principal role in hormone regulation. On the one hand, based on the “free hormone hypothesis,” the carrier proteins bind steroids and turn them to be biologically inactive (32). On the other hand, it was shown that both SHBG and CBG could bind to their target cells receptors and effect different molecular pathways and signaling such as apoptosis (33, 34). Several factors regulate SHBG expression in the liver including metabolic and hormonal factors such as glucose (35), thyroid hormone (36), and other factors such as the hepatocyte nuclear factor 4α (HNF-4α) (37), PPAR-γ (38), and p53 (14). Together, these findings point out that the liver, as the primary organ that produces and secrets these carrier proteins to the plasma (39), is the central regulator of steroid hormones bioavailability.

### Glucocorticoids

Glucocorticoids are vital endocrine regulators of homeostasis and adaptation to environmental variations. GC is widely used clinically as a potent anti-inflammatory and immunosuppressive agents (40). They act *via* genomic (transcriptional) and non-genomic mechanisms. Upon binding to their cognate intracellular receptor (GR), the complex is translocated into the nucleus, and regulates various genes transcription (40, 41). Cortisol, the main glucocorticoid secreted by the adrenal cortex, is essential for various cellular functions including the immune system, vascular tone maintenance, and more (42). Cortisol is a major player during stress and severe illness mainly by increasing cardiac output and vascular tonus and decreasing pro-inflammatory cytokines release (43, 44). Cortisol levels are under the control of the hypothalamus-pituitary-adrenal (HPA) axis through the adrenocorticotropic hormone (ACTH) and the corticotropin-releasing hormone (CRH) (42). Eighty percent of the circulating cortisol is synthesized mainly from the liver- secreted high-density lipoproteins (HDL) cholesterol. Thus, in severe low-density lipoproteins (LDL) cholesterol insufficiency, due to enzymatic metabolic errors or acquired cases, cortisol production can be impaired (45).

#### Glucocorticoids and Liver Diseases

Non-alcoholic fatty liver disease (NAFLD) is a metabolic disorder characterized by hepatic steatosis, namely, the presence of free fatty acids or triglycerides in the liver (46). Studies in animal models have shown that rodents on a high fat diet and chronically elevated GC had a dramatic exacerbation in the development of NAFLD (47). Similarly, patients with elevated GC levels, as in Cushing's syndrome, are prone to develop features of NAFLD (48). NAFLD, as well as other forms of liver diseases, can develop into cirrhosis, which is a late stage of scarring (fibrosis) of the liver. Cirrhosis characterized by low arterial pressure, circulatory failure, vasodilation, and increased production of cytokines (45). Cirrhotic patients were reported to suffer from adrenal insufficiency (AI), a condition where the adrenal glands cannot synthesize the adequate amount of cortisol (49). This is mainly due to their low cholesterol levels and to increased cytokines production such as TNF α, IL-6, IL-1, and endotoxin like lipopolysaccharide, that over stimulate and exhaust the HPA axis (50–53). The accumulating observations of the high prevalence (40–60%) of AI (54) in various stages of cirrhosis suggests that this disease may have a predisposition to AI, which may be a feature of liver disease *per se* (55–58). Indeed, using “conservative” diagnostic criteria, Marik et al. (59) reported a surprisingly high incidence of AI in a large cohort of critically ill patients with liver disease and coined the term “hepatoadrenal syndrome” to describe the association between adrenal dysfunction and liver disease. Rauschecker et al. demonstrated that AI is common, in patient with cirrhosis. This was done by using serum free cortisol for diagnosis, as protein abnormalities might affect interpretation of total serum cortisol levels (60).

It seems that end stage of liver disease may coexist with AI and on the contrary, excess glucocorticoids have been implicated in the pathogenesis of liver disease. Patients with NAFLD seem to have a subtle chronic activation of the hypothalamic pituitary adrenal axis leading to a state of subclinical hypercortisolism. Suggesting a bidirectional relationship between liver and the adrenal (61).

### Mineralocorticoids

Mineralocorticoids are hormones synthesized by the adrenal cortex that influence salt and water balance. Among the mineralocorticoids, the renin-angiotensin-aldosterone system (RAAS) is the principal hormonal system responsible for regulating cardiovascular, renal, and adrenal function and maintaining blood pressure and electrolyte balance (62).

Hepatocytes synthesis angiotensinogen (AGT) which is released into the bloodstream and transformed into angiotensin I and then to angiotensin II (ANG II), the main effector of this system. The significant biological actions of ANG II are mediated by binding two types of G protein coupled receptors, the angiotensin type 1 (AT1), and AT2 receptors. They can induce vasoconstriction and the release of aldosterone, which causes sodium and water reabsorption (63, 64). The AT1 and AT2 receptors are abundant in different tissues. AT1 receptors are expressed in hepatocytes, bile duct cells, hepatic stellate cells (HSC), myofibroblasts, and vascular endothelial cells (65). ANG II activates the AT1 receptors and induces HSC contraction and proliferation, oxidative stress, and inflammation responses, and endothelial cells dysfunction and growth (66). Moreover, in fibroblasts it upregulates TGF-β and collagen 1 gene expression (67–69). AT2 receptors were also found to be expressed in the liver (70) with possible anti-fibrogenic effects (71).

#### Mineralocorticoids and Liver Diseases

Renin-angiotensin-aldosterone system has an important role not only in the vascular system but also in different organs such as the liver. RAAS is implicated in various liver pathologies. Results of a prospective study suggest that patients with Hyperaldosteronism, a condition in which there is excessive secretion of aldosterone, frequently exhibit NAFLD (72). Indeed, ANG II was shown to be involved in cirrhosis-related portal hypertension (73, 74) and in the pathogenesis of insulin resistance and NAFLD, through its role in liver inflammation and fibrosis development (75, 76). Infusion of ANG II into mice was shown to upregulate TGF-β in normal rat livers (77), suggesting that upregulation of RAAS is sufficient to cause damage even in the absence of an underlying liver disease process (78). Thus, it is not surprising that due to the involvement of the RAAS system in liver disease, there is a growing interest in using RAAS inhibitors to treat NAFLD. Indeed, blocking RAAS by angiotensin-converting enzyme inhibitors and angiotensin receptor blockers reduced fibrosis in an experimental model of hepatic fibrosis (79).

#### Transcriptional Regulation of RAAS in Liver Diseases

Angiotensinogen has been suggested to be a key genetic determinant of RAAS. In experimental animal models a change, as little as 20%, in AGT expression levels are reflected by high blood pressure phenotype. *In vivo* studies have revealed that direct-repeat motifs (AGGTCA) within the human AGT gene promoter are functionally required for its expression in the liver (80). Examination of the human AGT gene-regulatory sequence revealed a single nucleotide polymorphism, resulting in two haplotypes. Haplotype I is associated with increased blood pressure, whereas, haplotype II is related to normal blood pressure. Interestingly, physiological changes, such as high fat diet, might change the transcriptional environment, and cause a modulation in AGT gene expression in a polymorphism dependent manner. Moreover, following high fat diet, increased levels of the transcription factors GR, HNF-1, STAT-3, and C/EBPβ were noticed in the liver and adipose tissues, leading to an increased expression of the AGT gene in liver tissue of haplotype I compared with haplotype II. These findings may have a significant clinical impact allowing the early identification and treatment of patients with the unfavorable haplotype (haplotype I) (81).

### Androgens

Androgens are the principal male sex hormones that regulate masculinizing effects and male sexual behavior. While the major circulating androgens are dehydroepiandrosterone (DHT), androstenedione, testosterone, and dihydrotestosterone, only testosterone and DHT can bind to the androgen receptor (AR) (82). Testosterone is considered to be the most significant androgen in humans (82), playing a significant role in controlling metabolism processes of carbohydrate, fat, glycogen, lipids, and cholesterol (83). Thus, it is not surprising that testosterone deficiency usually is characterized with liver diseases (83).

#### Androgens and Liver Diseases

Studies in men have shown an association between hepatic steatosis and low levels of serum testosterone (84). Moreover, mice with non-functional 5αR1, the enzyme that converts testosterone to DHT, exhibit hepatic steatosis (85, 86). Interestingly, ARs in male liver tissue have a critical role in maintaining lipid metabolism compared to female (83, 87). However, testosterone can also participate in the hepatic lipid deposition, independently of the classic AR, operating by regulating several critical lipogenic enzymes activity (88).

Androgens play a role in glucose and cholesterol homeostasis of the liver. Androgens regulate liver glucose homeostasis with gender differences; while in males high testosterone favors hepatic glucose metabolism in females it impairs (89). Several longitudinal studies have shown that low levels of testosterone independently predict the later development of type 2 diabetes or metabolic syndrome (89). Moreover, prostate cancer patients that are undergoing androgen deprivation therapy are under increased risk to develop diabetes (90, 91). Testosterone treatment in men with hypogonadism is associated with a significant reduction in fasting plasma glucose, HbA1c, fat mass, and triglycerides (92).

Low levels of testosterone have been associated with increased levels of LDL cholesterol, triglycerides, and with decreased HDL levels (83). Androgen replacement treatments resulted in decrease in serum LDL levels by enhancing liver cholesterol uptake, suppressing cholesterol removal, and promoting cholesterol storage (93).

Liver malfunction, manifested by the alteration in its metabolic homeostasis regulation, can pave the way to liver tumor development (94). Since hepatocellular carcinoma (HCC) is more prevalent in men rather than women, sex hormones might be involved in this malignant process. Indeed, higher levels of androgen signaling, reflected by higher testosterone levels, were found to increase the risk for HBV-related HCC in men (95). Moreover, long-term use of oral contraceptives and anabolic androgenic steroids can induce both benign and malignant hepatocellular tumors (96). Additional evidence comes from the fact that individuals with HCC express augmented levels of ARs in their tumor tissue and in the surrounding liver (97). These data support the notion that AR could affect HCC progression and that a combination of sorafenib, (kinase inhibitor drug approved for cancer treatment), together with AR inhibitors, might be a potentially improve treatment for patients with advanced HCC (98).

### Estrogens

Estrogens are the female principal sex hormones that regulate female reproductive, physiology, and sexual behavior. In humans, the most essential biologically relevant form of estrogens is the 17β estradiol (E2). While in premenopausal women E2 is mainly produced from cholesterol, in postmenopausal women it is primarily converted from testosterone by aromatase (99). E2 exerts its functions by binding to both the nuclear (ER) (ER-α and ER-β) and the membrane estrogen receptors (100). In addition to their expression in reproductive organs, ERs are also expressed to a lower extent in the liver (101–103).

#### Estrogens and Liver Diseases

Several studies have demonstrated that mimicking of menopause and reduction in estrogen signaling lead to insulin resistance, fatty liver, and dyslipidemia, as determined by decreased HDL and increased LDL and triglycerides (104–106). Indeed, while treatment with a specific ER-α agonist decreases fat mass and triglycerides, ER-α KO mice accumulate liver triglycerides and diglycerides (107–110). Furthermore, while estrogen deficiency causes steatosis, estrogen replacement decreases steatosis (111–113). Thus, it seems that estrogen together with ER-α plays a role in preventing liver malfunctioning. These data are in line with the observation that women in menopause are in a higher risk to develop NAFLD (114). Moreover, hormone treatment can reduce liver damage, as manifest by reduced plasma levels of liver enzymes (115).

#### Transcriptional Regulation Mediated by ER

Upon binding of E2 to ERs, they translocate into the nucleus where the receptors bind to specific genomic sequences. Interestingly, estrogen responsive elements (REs) are found in promoters and enhancers of liver genes. Genetic analysis revealed that more than 1,000 human liver genes have an expression based on sex bias (116). More than 40 lipids-related genes transcriptionally regulated by ER-α (117). Fascinatingly, this regulation is in tight coordination with the reproductive needs (118).

## p53 As a Major Factor in Hepatic Responses Regulation

p53 is a well-known tumor suppressor which plays a central role in cell fate determination through cell-cycle arrest, senescence, differentiation, and apoptosis, in response to various stress signals (119–121). Novel data suggest that p53 governs additional biological pathways besides its traditional role as a tumor suppressor. This includes the regulation of metabolism, as well as pathways that affect the cell microenvironment and regulation of general homeostasis (122). Therefore, it is not surprising that the liver, an organ that regulates many metabolic processes, and coordinates homeostasis, serves as a unique platform for p53 to perform its classical and non-classical activities, both in health and in disease (94). Indeed, changes in the ratio of the hepatic enzymes ALP, ALT, and AST which serves as indicators of liver failure (123), were observed in p53-KO mice. These observations suggest that physiological p53 is a main regulator of liver homeostasis (124). Upon stress, hepatic p53 is activated and acts as a double-edged sword. As a tumor suppressor, p53 functions locally by preventing the development of HCC (94), and distally by inducing apoptosis of breast cancer cells by the secretion of SHBG (14). In addition, activated p53 can induce the secretion of senescence-associated secretory phenotype (SASP) in hepatic cells. This induction leads to a reduction in the accumulation of fibrotic tissue and to the stimulation of immune surveillance, which maintains tissue homeostasis and suppresses cancer development (125, 126).

On the other hand, the p53 induced apoptosis in the liver may cause infiltration of inflammatory cells and may lead, in the long run, to steatohepatitis, cirrhosis, and even to HCC (94). Moreover, the hepatic p53 acts in non-cell autonomous fashion by affecting and altering the liver secretome in response to different signals. Thus, presenting a novel function for p53 in homeostatic regulation of metabolic processes within the liver (124). Some of these secreted factors are mainly related to cell migration, implying a cross-talk between a distal tumor and the liver (124). Furthermore, p53 was also found to be an important regulator of lipid homeostasis (127). Hepatic p53 was shown to bind directly to p53 REs within the chromatin and to induce the transcription of mainly three genes that represent the different aspects of lipid metabolism (Pltp, Abca12, and Cel) (12). Interestingly, it was suggested that p53 also binds p53 REs in the promoter of cytochrome P450 enzymes and regulates their expression. These include CYP3A43, CYP3A5, CYP3A7, CYP4F2, CYP4F3, CYP4F11, CYP4F12, CYP19A1, CYP21A2, CYP24A1, and CYP3A4 (13). The latter is known to be the main enzyme which participates in the biosynthesis of steroid hormones (20, 21). In a proceeding study, it was found that CYP21A2 that catalyzes the conversion of 17 alphahydroxyprogesterone to 11-deoxycortisol, the immediate precursor of cortisol (128), is also transcriptionally regulated by p53 (14).

Moreover, p53 was shown to induce the expression and secretion of both CBG and SHBG by directly binding to p53 REs in their promoter (14). These data are in line with the observation that p53-WT mice exhibit higher CBG expression compared with their p53-KO counterparts. It should be noted that p53-KO female mice are known to have difficulties in reproduction (129). Hence, it is intriguing to speculate whether the lower levels of CBG in p53-KO mice may influence the level of active or free androgens and lead to fertility difficulties.

p53 was shown to transcriptionally regulate aromatase, a key enzyme that converts androgens to estrogen (see also section Estrogen). A putative p53-binding element was found in intron 1 of the aromatase gene. Under high fat diet conditions, p53 KO male mice produced dramatically more testosterone than WT p53 mice, while the serum testosterone levels were not significantly different. The level of E2 was low in both groups of mice resulting in higher testosterone/E2 level in p53 KO male mice (130). Others have shown data suggesting that p53 is globally required for GC receptor nuclear export (131). As GCs exert their function in the nucleus this may place p53 as an important regulator of GCs function.

In sum, these data suggest that p53 is associated with the regulation of the endocrine system at large, and particularly within the liver, with high impact on steroid hormones.

## Concluding Remarks

**Figure 1:**
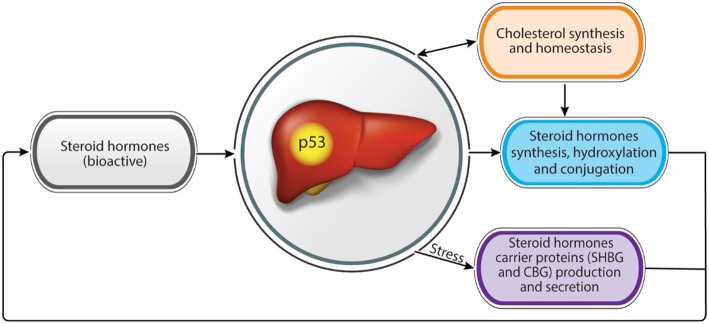
Steroid hormones homeostasis regulation by the liver. Functional intertwine exists between the liver and the steroid hormones. While the liver contributes to adequate levels of bioactive steroid hormones, through the modulation of synthesis and bioactivity, steroid hormones contribute to proper liver functions. p53 has a dual effect in regulating both steroid hormone levels and bioactivity as well as in maintaining liver hemostasis suggesting a role as a potential regulator in this axis.

Nowadays, population exposure to environmental steroid hormones, endocrine disruptor chemicals, and the increasing repertoire of medicines used by the public has an increasing impact on steroid hormones levels and activities as well as liver functions, leading to various liver diseases and endocrine syndromes. A comprehensive understanding of the molecular mechanisms underlying these diseases may help to prevent and treat them. An apparent bidirectional link exists between steroid hormones over secretion or insufficiency and liver metabolic or functional disease. Moreover, the liver and p53 are involved in steroidogenesis and steroid hormones homeostasis and balance. This notion may place p53 as a potential regulator in the liver/steroid hormone axis, maintaining homeostasis and preventing diseases (Figure 1).

## Author Contributions

MC-N, RA-G, and EO wrote the manuscript. VR edited and reviewed the manuscript.

### Conflict of Interest Statement

The authors declare that the research was conducted in the absence of any commercial or financial relationships that could be construed as a potential conflict of interest.
